# Sensitivity Analysis of an ENteric Immunity SImulator (ENISI)-Based Model of Immune Responses to *Helicobacter pylori* Infection

**DOI:** 10.1371/journal.pone.0136139

**Published:** 2015-09-01

**Authors:** Maksudul Alam, Xinwei Deng, Casandra Philipson, Josep Bassaganya-Riera, Keith Bisset, Adria Carbo, Stephen Eubank, Raquel Hontecillas, Stefan Hoops, Yongguo Mei, Vida Abedi, Madhav Marathe

**Affiliations:** 1 Network Dynamics and Simulation Science Laboratory, Virginia Bioinformatics Institute, Virginia Tech, Blacksburg, Virginia, United States of America; 2 Nutritional Immunology and Molecular Medicine Laboratory, Virginia Bioinformatics Institute, Virginia Tech, Blacksburg, Virginia, United States of America; 3 Center for Modeling Immunity to Enteric Pathogens, Virginia Bioinformatics Institute, Virginia Tech, Blacksburg, Virginia, United States of America; 4 Department of Biomedical Sciences and Pathobiology, Virginia-Maryland Regional College of Veterinary Medicine, Virginia Tech, Blacksburg, Virginia, United States of America; 5 Department of Computer Science, Virginia Tech, Blacksburg, Virginia, United States of America; 6 Department of Statistics, Virginia Tech, Blacksburg, Virginia, United States of America; National Research Council of Italy (CNR), ITALY

## Abstract

Agent-based models (ABM) are widely used to study immune systems, providing a procedural and interactive view of the underlying system. The interaction of components and the behavior of individual objects is described procedurally as a function of the internal states and the local interactions, which are often stochastic in nature. Such models typically have complex structures and consist of a large number of modeling parameters. Determining the key modeling parameters which govern the outcomes of the system is very challenging. Sensitivity analysis plays a vital role in quantifying the impact of modeling parameters in massively interacting systems, including large complex ABM. The high computational cost of executing simulations impedes running experiments with exhaustive parameter settings. Existing techniques of analyzing such a complex system typically focus on local sensitivity analysis, i.e. one parameter at a time, or a close “neighborhood” of particular parameter settings. However, such methods are not adequate to measure the uncertainty and sensitivity of parameters accurately because they overlook the global impacts of parameters on the system. In this article, we develop novel experimental design and analysis techniques to perform both global and local sensitivity analysis of large-scale ABMs. The proposed method can efficiently identify the most significant parameters and quantify their contributions to outcomes of the system. We demonstrate the proposed methodology for ENteric Immune SImulator (ENISI), a large-scale ABM environment, using a computational model of immune responses to *Helicobacter pylori* colonization of the gastric mucosa.

## Introduction

Sensitivity analysis (SA) is the study of the impact of different parameters in the outcomes of a system [1, 2]. SA has been used in many scientific fields to highlight important data, optimize the design of a system, simplify models, and rank the influence of various parameters on a given system [3–6]. SA can be performed in a local or global context. Local SA examines the effect of deviations of a parameter (within its range), on system outcomes around a base setting [7], i.e., only one parameter is changed while all others remain fixed. Global SA evaluates the entire parameter space to determine all of the system’s critical points [3, 8]. Both statistical and deterministic methods can be used for SA [2, 9]. Other approaches for SA include variance decomposition [10], response surface [11, 12], and sampling methodologies [13]. The choice of method depends on the purpose and the system under study. Typically the systems under review are computationally expensive and hence SA becomes very challenging.

ABM is a computational technique where the behavior of individual agents or groups of agents is encoded by simple rules, and the outcomes are observed at the system scale. ABM is a widely used technique in different areas of study such as computer science [14], economics, biology, ecology, and social phenomena. Popularity of ABM is also due to its intrinsically spatial component [15] and the fact that the system allows integration of different experimental data into one *in silico* experimental system very easily [16]. In fact, this modeling framework can be used to intuitively map biological (or social) phenomena and at the same time capture some of the inherent stochastic nature of these systems without an added layer of complexity [15, 17–20]. Furthermore, ABM offers modeling intuitive implementation of interactions (e.g. binding of molecules) where rules that describe the interactions are often taken from existing literature. A concrete example: a large body of studies have successfully used ABM to simulate dynamics of inflammation [21–26]. ABM has become even more popular during the past decade with the exponential growth of technological and computational power. For instance, in 2008, agent based modeling and simulation studies were used to explore the development and resolution of virtual infections in a unique way [27, 28]; the study was fundamental in the identification of key parameters capable of inducing clearance, persistent infection, or even death. For a comprehensive review of ABM applied to host-pathogen systems, the reader is referred to a review by Bauer *et al*., [29], where authors describe various models but also discuss some of the limitations and challenges.

We use a formal mathematical framework called graphical dynamical systems (GDS) [30, 31] (see S1 Text for definition) to represent the agent based models studied here. This representation elucidates the formal aspects of the model as a discrete and finite dynamical system. GDS is a widely used framework for capturing distributed, dynamical systems such as social contact graphs [32], urban traffic and transportation [33], epidemics [30], dynamics of biological systems [34], and gene annotation [35]. The configuration space of an ABM, represented as a GDS *G*, can be conveniently viewed as a discrete Markov chain *M*. Each node in *M* is the state vector of the node states in *G*. A directed edge from node *A* to *B* in *M* with label *p* denotes the probability of transition from *A* to *B*. The nodes of *M* are also referred to as the state space of *M*. For example, a complex ABM comprised of one million agents, each of these agents having six possible states, results in a Markov chain with a state space consisting of 6^10^6^^ different configurations. Note that the Markov chain is represented succinctly by the ABM. The chain is exponentially larger than its representation. Straightforward methods for global sensitivity analysis on such large chains are unlikely to scale. It is worth pointing out that the proposed sensitivity analysis are not restricted to GDS. It is rather a general methodology applicable to a wide range of complex simulation models. However, we believe having a good understanding of the presented system can be beneficial as it can provide a direct example of a complex system where such a method can be used. The size of the state space makes the experimental design of interest. If the state space was small, one could instead use many other designs that can exhaustively cover the range of parameter values.

In this paper, we perform sensitivity analysis on a complex ABM named ENteric Immunity SImulator (ENISI) [36–38]. ENISI is a modeling environment for studying the inflammatory and regulatory immune responses in the gut. During the development of ENISI, three versions were released; ENISI HPC [39], ENISI Visual [40], and ENISI MSM [41, 42]. ENISI HPC focuses on scalability by implementing a parallel simulation framework. ENISI Visual focuses on visualizations. ENISI MSM, a multiscale modeling framework, focuses on the integration of heterogeneous modeling technologies. The SA method presented here can therefore be utilized directly for the three different technologies in addition to being valuable to other ABM systems. The novelty lies in the choice of appropriate designs that enable statistical methods to explore parts of the Markov chain in a computationally efficient manner and still guarantee certain statistical properties. We use orthogonal arrays and variation-based sensitivity analysis to achieve this, which is discussed in detail in subsequent sections.

Developing novel approaches for sensitivity analysis of agent-based models enhances understanding of the influence of different input parameters and their variations on model outcomes and has the potential to improve the parameter estimation process by making it more systematic and streamlined. The objective of the sensitivity analysis is to identify the greatest and least significant parameters in the model, and to quantify how parameter uncertainty influences outcomes. Analytically exploring the behavior of such systems, even if model outcomes mirror reality, is challenging due to the large number of parameters [2]. Sensitivity analysis is also important for understanding the relationships between input and output variables, for testing the robustness of the system, and for identifying errors in the model.

### ENISI: an agent-based modeling environment

ENteric Immunity SImulator (ENISI) is a modeling environment for studying inflammatory/effector and regulatory immune responses in the gut [36–38]. ENISI encodes immune pathways as an agent-based model representing individual cells that participate and interact with other cells in the gastrointestinal tract. The individual cells are distributed spatially to four *locations*: lumen, epithelial barrier (EB), lamina propria (LP), and gastric lymph node (GLN). *Locations* are further divided into many discrete patches called *sublocations*. A *sublocation* is defined as the maximum volume at which a cell can be assumed to be in contact with all other cells. *Sublocations* represent the discrete region where cells remain and interact. Each cell moves between *sublocations* according to its own *schedule*. A cell can have its own movement *schedule* assigned by phenotype or *state* and *location*. Within a *location*, cells move between the *scheduled* *sublocations* at short time intervals, which represents random movement and produces a dynamic contact network. This random movement, which does not favor movement to adjacent *sublocations*, is added because of an assumption that cells are moving at a much faster rate than the time period for *sublocation* update (30 minutes). Therefore, the probability of an individual cell having traveled far from the current *sublocation* is the same as the probability of being in a nearby *sublocation* after the 30 minute interval.

### Parameters

The ENISI model of immune responses to *H. pylori* has a set of 25 independent modeling parameters. These modeling parameters include transition probabilities, constant factors of group-cell interactions, stimulation/inhibition factors, and migration rates. Each of the 25 parameters is a continuous input variable within corresponding parameter ranges. Parameter values are obtained from the literature whenever available or estimated during model development [43–48]. A complete list of parameters along with a short description, range, and default value is shown in Table 1.

**Table 1:** A complete list of parameter values for ENISI.

Parameter	Description	Range	Default Value
*α*_*T*_	probability of resting T cell stimulation	[0, 1]	1
*p*_17_	probability of resting T cell stimulation to *Th*1 vs. *Th*17 by *eDC* or *M*1	[0, 1]	0.5
*α*_*nTreg*_	probability of resting *nTreg* stimulation	[0, 1]	1
*ν*_*T*_	fraction of active T cells that become memory T cells	[0, 1]	0.1 [47]
*a*_1_	co-efficient of $\nu_{12}={\left(\frac{a_{1}R}{a_{1}R+i_{1}N}\right)}^{y_{1}}$ (Pr(*M*1 → *M*2)) for activators	[0, 10]	1
*i*_1_	co-efficient of *ν*_12_ for inhibitors	[0, 10]	1
*y*_1_	exponent of *ν*_12_	[0, 4]	4
*a*_2_	co-efficient of $\nu_{21}={\left(\frac{a_{2}R}{a_{2}R+i_{2}N}\right)}^{y_{2}}$ (Pr(*M*2 → *M*1)) for activators	[0, 10]	1
*i*_2_	co-efficient of *ν*_21_ for inhibitors	[0, 10]	1
*y*_2_	exponent of *ν*_21_	[0, 4]	4
*a*_*r*_	co-efficient of $\nu_{r17}={\left(\frac{a_{r}N}{i_{r}R+a_{r}N}\right)}^{y_{r}}$ (Pr(*iTreg* → *Th*17)) for activators	[0, 10]	1
*i*_*r*_	co-efficient of *ν*_*r*17_ for inhibitors	[0, 10]	1
*y*_*r*_	exponent of *ν*_*r*17_	[0, 4]	4
*a*_17_	co-efficient of $\nu_{17r}={\left(\frac{a_{17}R}{a_{17}R+i_{17}N}\right)}^{y_{17}}$ (Pr(*Th*17 → *iTreg*)) for activators	[0, 10]	1
*i*_17_	co-efficient of *ν*_17*r*_ for inhibitors	[0, 10]	1
*y*_17_	exponent of *ν*_17*r*_	[0, 4]	4
*ν*_*BM*_	probability that commensal bacteria induces inflammatory phenotype in macrophages	[0, 1]	0
*ν*_*BD*_	probability that commensal bacteria induces inflammatory phenotype in dendritic cells	[0, 1]	0
*ν*_*Bs*_	probability that commensal bacteria induces inflammatory phenotype in ‘sampling’ dendritic cells	[0, 1]	0
*ν*_*EC*_	probability that *EC* transitions to *pEcell* upon contact within flammatory factors	[0, 1]	0.05
*ν*_*EB*_	probability that *EC* is damaged by microbial toxins	[0, 1]	1 [48]
*β*_*r*_	ability of commensal or inflammatory bacteria to induce chemoattractant expression in epithelial cells	[0, 1]	1
*μ*_*ce*_	probability that *pEcell* is killed by inflammatory factors	[0, 1]	0.5
*β*_*d*_	relative amount of microbicide secreted by *pECell*, *pEC*_*noR* in response to commensal or inflammatory bacteria	[0, 1]	1
*μ*_*M*1_	ability of *M*1 to eliminate bacteria	[0, 1]	1

### System output

The ENISI model under study has eight types of immune cells and bacteria (epithelial cells, tolerogenic bacteria, commensal bacteria, dendritic cells, sampling dendritic cells, macrophages, CD4+ T cells and natural T-regulatory cells) involved in the gastric immune responses to *H. pylori*. Each individual cell has many different functional states called phenotypes. There are a total of thirty different phenotypes in this model. Among those, activated/effector CD4+ T cells and macrophages are the most interesting cell types for the disease model under study. Furthermore, there are four different tissue sites modeled in ENISI: lumen, epithelial barrier (EB), lamina propria (LP), and gastric lymph node (GLN). ENISI provides the number of cells of each phenotype grouped by *location* as the system output. A complete list of cell types is available in [36].

### Aims and challenges

The aims of this study are to (1) evaluate the influence of parameters on model outcomes, (2) rank the parameters based on their global influence, and (3) refine the ABM based on the result of the SA of the parameters. In this study, we used ENISI to recreate an experimental infection of an individual mouse with *Helicobacter pylori*, a bacteria that colonizes the human stomach chronically causing disease in 15% of carriers. The particular strain in this experiment is the Cag Pathogenicity Island (CagPAI)+ *H. pylori* strain 26695 [36–38]. However, it is important to indicate that this analysis can be applied to an array of complex biological networks including the model of CD4+ T cell differentiation [49–51], network dynamics of T helper 17 induction and differentiation [52], and network of interactions between mucosal immunity and the gut microbiome during *Clostridium difficile* infection [53].

The key challenges of the study lie in the size and number of parameters of the model and the computational cost associated with the stochastic nature of the process. A simulation in ENISI consists of a magnitude of 10^7^ individual agents. There are thirty different types of agents and a few hundred individual rules. On a modern high performance computing cluster of 48 nodes, a single run of the simulation takes about ninety minutes on average. Because of this limitation, the runtime of multiple simulations must be taken into consideration. Each of the 25 parameters is considered to be continuous; however, if each of the 25 parameters takes only four different values, then a full factorial experimental design would require 4^25^ runs, which is not possible in terms of the system’s runtime. To alleviate this problem we propose a novel experimental design with an economic number of simulations.

## Materials and Methods

### Related Works

Because sensitivity analysis can be an effective tool for systematically identifying the most and the least significant parameters, it can provide valuable information on the robustness of biological systems. Summer *et al*., [54, 55] implemented a SA method by exploring a group-based procedure, where SA is applied to groups of parameters. The result of this analysis can be used to identify the components of the model that are most important in determining the model behavior. This method was applied to a composite model of blood glucose homeostasis that combines models of processes at the sub-cellular, cellular and organ level to describe the physiological system [54–56]. Furthermore, using a simple ABM to assess different methodologies for SA, van Voorn *et al*. [57] highlighted the importance of using a local as well as global method; while the local sensitivity can capture tipping points, the global approach can provide a summary measure of parameters. Along the same line of research, Schouten *et al*., [58] compared two methods of SA–local and global and concluded that a mixed approach of SA can lead to a better understanding of model behavior. In the global SA, the authors used an approach based on Monte Carlo sampling of parameters. In a recent study, Ligmann-Zielinska *et al*. [59] argue that SA as well as uncertainty analysis (UA) are important elements in quantification of model output variability and its sensitivity to inputs. On the basis of this argument, authors utilized variance decomposition of the ABM output to demonstrate how an integrated quantitative UA in combination with SA can be used in ABM development. Related analyses include the work done by Parry *et al*., [60] using a Bayesian approach for SA for the identification of sensitive elements, and work done by Fonoberova *et al*., [61] using a variance-based as well as derivative-based global SA in the identification of key elements for model reduction. In this paper we consider both global and local sensitivity analysis of ABM using ANOVA and discuss the most and the least significant parameters which could be used for model reduction as in [61]. Though there are numerous instances of SA applied to ABMs, few attempt to classify the behavior of a parameter. We used main effect plots to classify the behavior of input parameters into four classes in a novel fashion that provides both visual representations of and statistical insights into a parameter. Our choice of four levels reduces the computational costs associated with large scale ABM while regaining valuable insights into the parameter. These statistical analyses along with sparse orthogonal array based experimental design provide a concrete framework to perform SA on any agent based models.

### Global sensitivity analysis

In global sensitivity analysis we study the behavior of input parameters on the variation of the model output. Not every modeling parameter has equal effects on system outcomes. Some parameters play more significant roles than others. The goal is to quantify the significance of the parameters based on some measurement.

### Global versus Local sensitivity analysis

A local sensitivity analysis addresses sensitivity relative to change of a single parameter value, while a global analysis examines sensitivity with regard to the entire parameter distribution.

Global sensitivity analysis focuses on the variance of model outputs and determines how input parameters influence the output parameters. It is a central tool in sensitivity analysis since it provides a quantitative and rigorous overview of how different inputs influence the output.

Local sensitivity analysis focuses more on a single input’s behavior while other parts remain the same. It is narrow in this aspect as the effect of input parameter is not measured for settings other than the base. Global sensitivity analysis is often preferred when possible, due to its greater detail, but for a large system it is time inefficient to run a model. Local sensitivity analysis method can be preferred because it requires less computation.

#### Choosing suitable model output

To measure the sensitivity of the model with respect to modeling parameters we need to select suitable output variables. One challenge regarding a complex system like ENISI is that it has many output variables. One of the outputs provided by ENISI is the number of cells of each cell type in every *location*. There are about 30 total possible phenotypes for the eight types of cells modeled in ENISI. Measuring sensitivity against all the model outputs is difficult and time consuming. In order to conduct the analysis more effectively, we chose nine output variables that are the primary concerns of immunologists [62, 63]. In this study, three phenotypes of T cells are considered: *Th*1, *Th*17, and *iTreg* in the *locations* LP and GLN as the primary output of the system. Three types of macrophages: *M*1 (classically activated, prone to promote inflammation), *M*2 (alternatively activated with regulatory and pro-resolutory functions) and *M*0 (precursor of *M*1 and *M*2 macrophages) in the LP *location* are also considered. A list of these nine outputs is provided in Table 2.

**Table 2:** System Output Phenotypes.

State	Description
*Th*1_*LP*	Active T helper-1 cell in lamina propria
*Th*17_*LP*	Active T helper-17 cell in lamina propria
*iTreg*_*LP*	Induced T regulatory cell in lamina propria
*Th*1_*GLN*	Active T helper-1 cell in gastric lymph node
*Th*17_*GLN*	Active T helper-17 cell in gastric lymph node
*iTreg*_*GLN*	Induced T regulatory cell in gastric lymph node
*M*0_*LP*	Undifferentiated macrophage in lamina propria
*M*1_*LP*	Activated inflammatory macrophage in lamina propria
*M*2_*LP*	Activated regulatory macrophage in lamina propria

ENISI models the immune response and pathology at the gastric mucosa during *H. pylori* infection. In the stomach *H. pylori* is mainly found in the mucus layer lining the epithelial cells, and a small fraction is present in the lamina propria (LP). Cells participating in the immune response to the bacteria, such as CD4+ T cells and macrophages, are found in the lamina propria. Cells in the gastric lymph nodes (GLN) region also play an important role in the induction of the immune response. Different phenotypes of T cells and macrophages play vital roles in the inflammation and immuno-regulation process [62–64]. For this reason we chose regulatory T cells, T helper 1, and T helper 17 cells in both LP and GLN sites and *M*0, *M*1, and *M*2 macrophages in the LP site as the output variables. We also have biological lab data available for these cell types for model calibration.

#### Choosing input parameter values

In this study, the value of each parameter has continuous values with a fixed range as shown in Table 1. In order to conduct variation-based sensitivity analysis [1], a common approach is to choose several levels for each parameter such that the analysis of variance can be performed in a rigorous fashion. In this work, we consider each parameter to have four discrete values (levels) in increasing order: Level 1 to Level 4. The exact value for each level is dependent on the corresponding parameter range from Table 1. Exact values for the four levels of each parameter are given in S1 File. We observed that most of the parameter values in the default calibrated settings tend to take values lower in the range. This is reflected in our choice of values for the levels. With four levels in each parameter, each input parameter has three degrees of freedom when conducting variation-based analysis. In a simple word, one can fit a polynomial function for each parameter with degrees three. Thus, one can study the significance of the linear, quadratic, and cubic effects of the input parameter on the output. It provides flexibility on investigating how the input parameter affects the output for the agent-based simulation model. If one only chooses two levels for each parameter, then only the linear effect of each parameter could be studied [65]. It is worth noting that for a different agent based model, it may not be feasible to have the same number of levels for each parameter. In this situation, we would consider a mixed-level experimental design [66] to address this challenge. We also like to remark that the choice of four levels provides enough information to extract linear and non-linear effects in this study. However, when having fewer parameters and high computational power, modelers can further increase the number of levels to enrich the predictive power of the analysis.

### Design of Experiments

The results of global sensitivity analysis can be dependent on the distribution of input variables [67]. In this work we have performed global sensitivity analysis on ENISI using 25 input factors. Each experiment has 250 time steps. Each simulation time step represents six hours in real world. For each factor, there are four different levels. Note that if one conducts a full factorial design for the 25 factors, it requires 4^25^ = 1.126 × 10^15^ runs of experiments. Running one experiment in ENISI can take ninety minutes on average. Clearly, it is not possible to consider all possible combinations of parameter settings. A sparse design with economic run size is needed to conduct the sensitivity analysis.

As an effective statistical technique for sensitivity analysis, design of experiments (DOE) provides a way to carry out simulation experiments systematically that takes into account parameter interactions [68, 69]. By using experimental design, one can systematically bring statistical aspects into the analysis of results [70, 71]. It helps understand the relationships between input parameters and outputs in the model. DOE originates from real world experimentation in agriculture and engineering, and the techniques can be transferred to experiments with computer simulation such as agent-based models [72]. In the context of agent-based models, sensitivity analysis using DOE can uncover details about model behavior, help to identify the relative importance of inputs, and provide a common basis for discussing simulation results.

**Fig 1:**
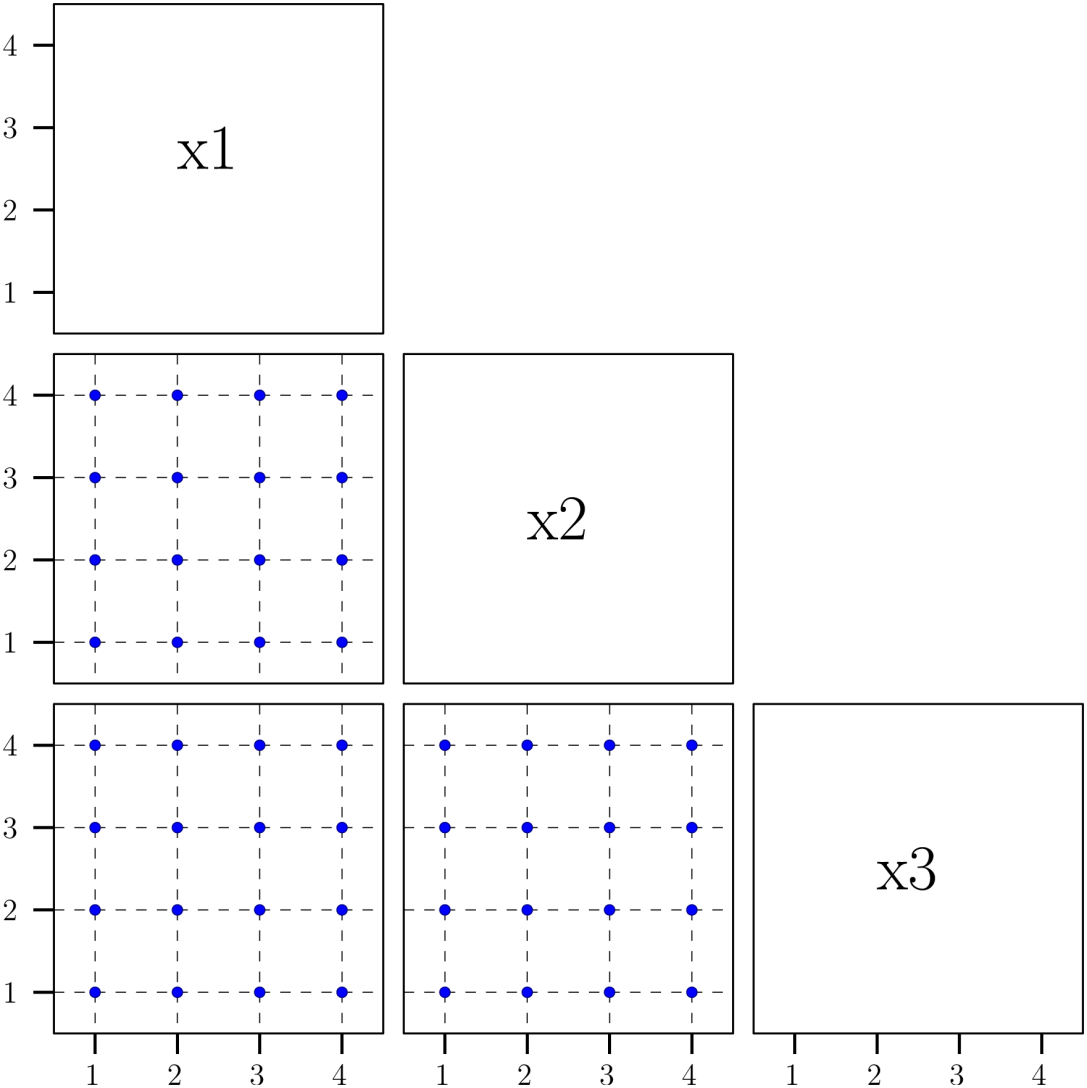
Illustration of orthogonal array experimental design with 128 runs and 25 factors. projecting the design points onto any two-dimensional subspace, the projected points always form a full factorial (4 × 4) with 8 replications for each level of combination.

In traditional DOE a fractional factorial design, which considers a fraction of all possible level combinations, is often used for the analysis [73]. However, for a problem with a large number of parameters, the number of runs of the fractional factorial design is still large. To overcome the drawback of using a fractional factorial design, we utilize an experimental design strategy using an orthogonal array [65, 68, 73] to obtain a sparse design with desirable properties. The designs of orthogonal arrays often have small run sizes, but can accommodate all possible level combinations for at least any two columns. Specifically, an orthogonal array (OA) with strength *t* in experimental design is a design matrix such that for every *t* column, the possible distinct rows all appear the same number of times. That is, when projecting the designs in OA onto any subspace with dimensionality *t*, the coverage of parameter space is the same as that using full factorial design in *t*-dimensional space. Such a property maintains a good balance of level assignment for each factor. A random design will not guarantee this property [65, 68, 73, 74]. Thus, sensitivity analysis based on a design of orthogonal array enables the comprehensive investigation of parameters without dramatically increasing the number of simulation runs. Designs of orthogonal array have been widely used in many engineering applications [74] and sensitivity analysis of computer models [75]. For the sensitivity analysis of ENISI, we consider an orthogonal array of 128 runs on 25 factors with strength 2 (see S2 File for the complete design). The details of this design can be seen in [74]. This design has the following attractive properties: (i) projecting the design points onto any factor, there are exactly 16 replicates for each level; (ii) projecting the design points onto any two variables (i.e., any two columns of the design matrix), it is a full factorial (4 × 4) with 8 replications for each level combination. Fig 1 illustrates an OA design with 128 runs in 25-dimensional space. When it project points to a three-dimension subspace (labeled as *x*_1_, *x*_2_, and *x*_3_), the projected points consist of full factorial design in any two dimensional space of (*x*_1_, *x*_2_), (*x*_1_, *x*_3_), and (*x*_2_, *x*_3_). A random design will not be able to achieve this property. Hence, the proposed design is sparse in the sense that there are only 128 points in a 25-dimensional space, while the design points are well spread out with good properties. Therefore, the proposed design enables the effective study of the main effect of any factor.

#### Experiments and Analysis

We used a high performance computing cluster of 48 Intel Sandy Bridge nodes. Each node consists of two dual-socket Intel Sandy Bridge E5-2670 2.60GHz 8-core processors (16 cores per node) and 64GB of 1600MHz DDR3 RAM. The nodes are linked by QLogic QDR InfiniBand interconnects. For the MPI based implementation of our algorithms, we used the MPICH2 (version 1.7), which is optimized for QLogic InfiniBand cards. For each experiment we used 48 processors. On average it took ninety minutes to complete one simulation. Our cluster allowed us to run around five simulations in parallel.

According to the design of experiment we selected 128 sets of distinct parameter settings. As the model is stochastic in nature, there is inherent uncertainty in the output even though all the input parameters are kept same. Therefore we repeated the experiment for every parameter settings 15 times. In total 128 × 15 = 1920 simulation runs were conducted for global sensitivity analysis. A summary of the experiments is given in Table 3.

**Table 3:** Summary of experimental design.

Input size	10^7^ individual cells
Number of parameters	25
Number of observed output variables	9
Number of experiments	128
Number of replicates	15

## Results

Aim 1: Determine the influence of parameters by performing global sensitivity analysis using partial factorial experiments. An analysis of variance (ANOVA) is also performed to compute the level of significance for every parameter. Aim 2: Ranking the parameters (from greatest to least effect) from Aim 1, followed by studying the underlying causes with biological validation. Aim 3: Fine tuning of parameter values by applying local sensitivity analysis to the model.

### Global sensitivity analysis determines the influence of a modeling parameter

The first attempt is to determine if there are any patterns of parameter influence on the system. To achieve this, we performed global sensitivity analysis of the system by following the design described in the “Materials and Methods” section. In this design each modeling parameter takes four monotonically increasing discrete values called “Levels.” Thus every parameter has four values: Level 1 to 4.

**Fig 2:**
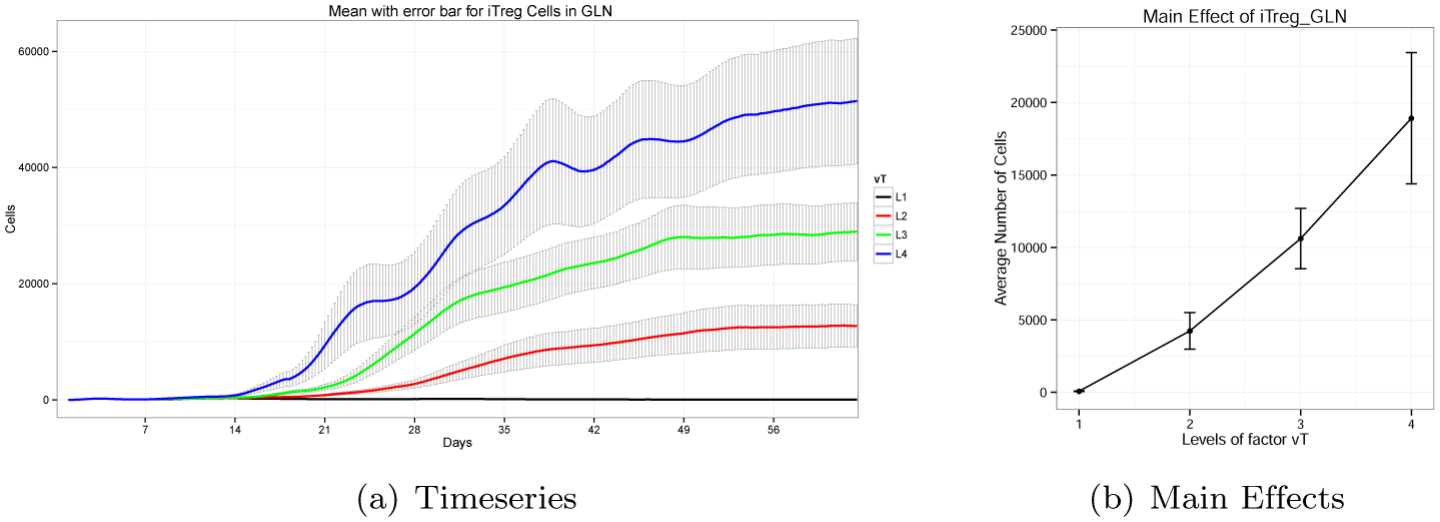
Timeseries and Main Effects of *iTreg* cells in gastric lymph node for Parameter *v*_*T*_.

ENISI provides the total number of agents per phenotype in each time step as the system output. Typically these output are represented as time series plots, where the *x* and *y* axes represent time step and the number of cells respectively. To quantify the influence of any parameter on the model output, we generated time series plots for each input parameter. The time series plot for a parameter has four curves, each belonging to a distinct parameter Level. As shown in the design diagram, a total of 128 experiments with different parameter settings were performed, where each experiment was replicated 15 times. According to the experimental design, each parameter has 32 distinct experiments per level. Hence, each curve of the time series plot has 32 × 15 = 480 simulation runs. The average of the runs are drawn along with the associated error bars in each curve. For example, Fig 2(a) shows the time series curves (along with error-bars) of *iTreg* cells in the gastric lymph node (GLN) for modeling parameter *v*_*T*_.

Though these curves provide intuitive visual representation, comparisons between curves are difficult. These curves also fail to show the changes of system output when the levels of the corresponding modeling parameter are changed. Therefore, we used a different representation called a “main effect” plot (see Fig 2(b)). In a main effect plot, the x-axis represents the levels of the modeling parameter. The y-axis represents the average number of cells per level over the entire time period. Note that each experiment has 250 time steps and is replicated 15 times for each level. The average number of cells per level is determined from the average of all these experiments over the 250 time steps for the main effect plot. In this way, a time series curve is converted into a scalar value. Fig 2(b) shows the main effect plot of *iTreg* phenotype in the gastric lymph node (GLN) for parameter *v*_*T*_. From this, it is easy to see that with increasing levels of *v*_*T*_, the average number of *iTreg* phenotypes in GLN increases in a monotonic fashion. Though the pattern of this particular parameter is clear, there exist other patterns of main effect plots that are more challenging to interpret. We will address these patterns herein.

#### Main effect plots can be classified into four distinct patterns

**Fig 3:**
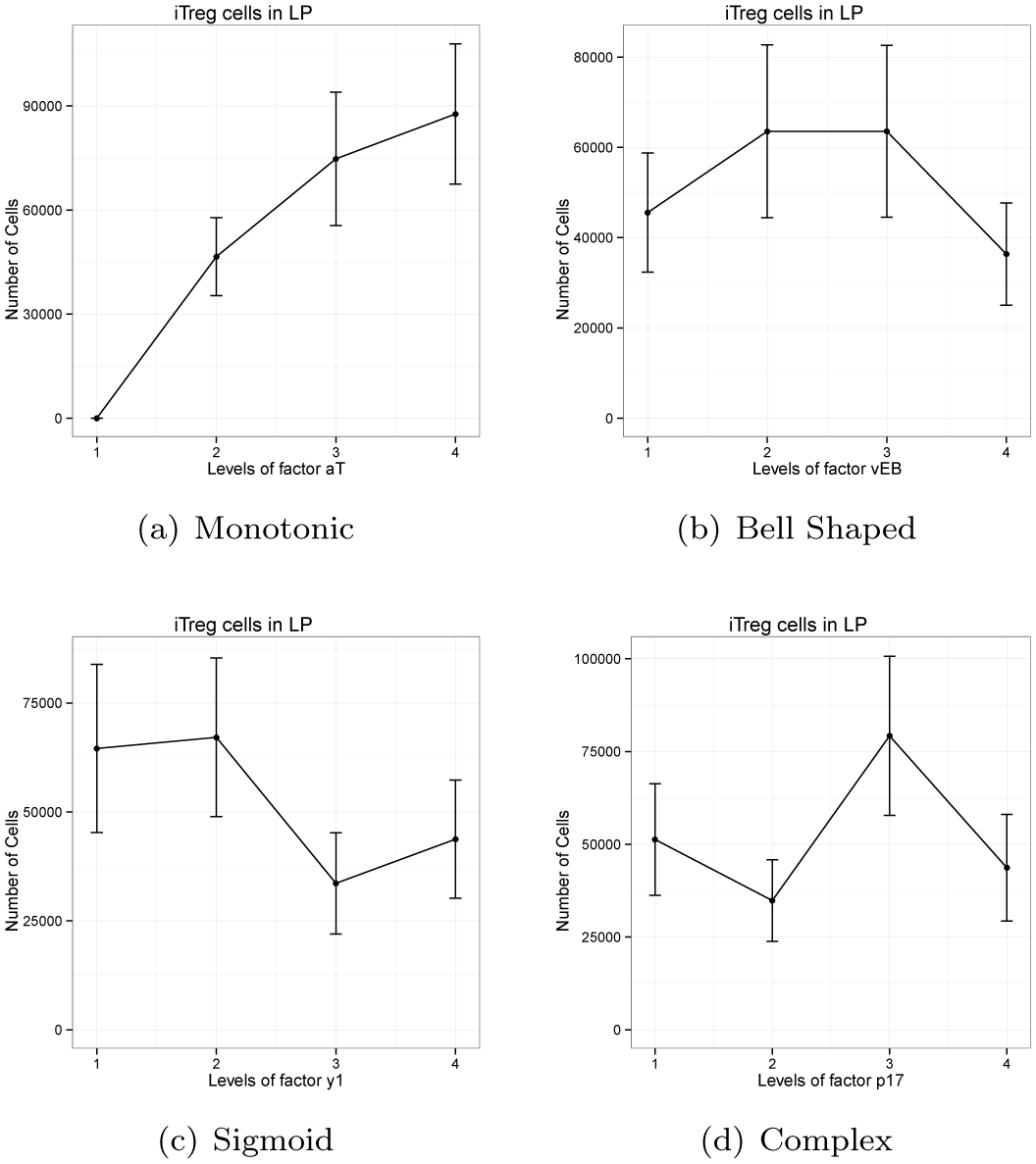
Four distinct patterns for *iTreg* cells in the LP.

The analysis of the main effect plots for every output reveals four distinct patterns. They are: 1) monotonic, 2) bell shaped, 3) sigmoid, and 4) complex patterns. These are shown in Fig 3.

The monotonic pattern in Fig 3(a) shows that increasing the values of the parameter *a*_*T*_, results in the average cell count going up (or down) monotonically. The bell shaped pattern (Fig 3(b)) shows the average number of cells in the middle of the parameter range are significantly higher (or lower) than the extreme ends of the parameter range. The sigmoid pattern is shown in Fig 3(c). Notice that between Level 2 and Level 3 the average number of cells are changed dynamically but Level 2 & Level 1 and Level 3 & Level 4 have almost the same average number of cells. Thus, sigmoid patterns can be considered a switch between two consecutive parameter levels. The complex pattern in Fig 3(d) shows the average number of cells vary significantly between any two consecutive parameter levels, thereby forming a zig-zag shaped complex pattern.

#### Monotonic main effect patterns have significant influence over the system

**Fig 4:**
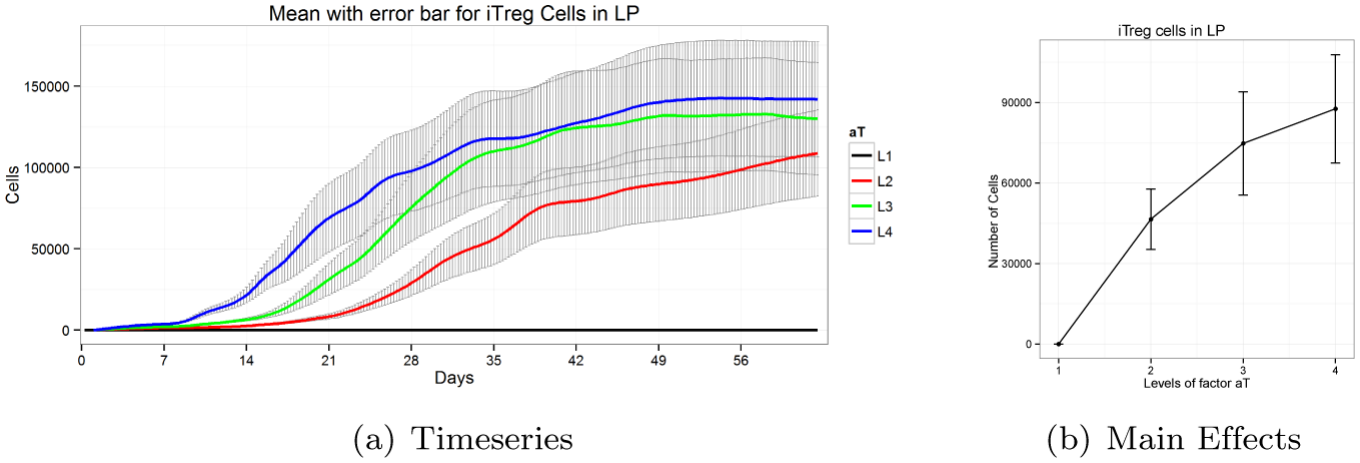
Timeseries and main effects of *iTreg* cells in lamina propria for parameter *a*_*T*_ exhibits monotonic pattern.

One of the prominent effects of a parameter on a complex system is monotonic in nature. In this case, with the increase of a parameter we observe monotonic increase or decrease of the model output. Fig 4 shows the time series and main effect plot of induced regulatory T cells in the gastric lamina propria for the parameter *a*_*T*_. Parameter *a*_*T*_ represents the stimulation rate of naïve T cells into active cells. With the increase of *a*_*T*_ the population of Treg cells is increased. From the main effect plot, we can clearly observe that with the increase of parameter *a*_*T*_ = 0 (Level 1) to *a*_*T*_ = 1 (Level 4), the population of Treg cells are also increased monotonically. Monotonic relationships of parameters are very common in immune responses and play crucial role in biological systems. Indeed, experimental data published in [64] shows an increase in numbers of the regulatory CD4+ T cells (Treg) subset during *Helicobacter pylori* infection. Based on these results, we could partially attribute the upregulation of the regulatory T cell population, among other effects, to the upregulation of the rate of T cell activation due to cytokine signaling [64].

#### Bell shaped patterns reveal interdependency among modeling parameters

**Fig 5:**
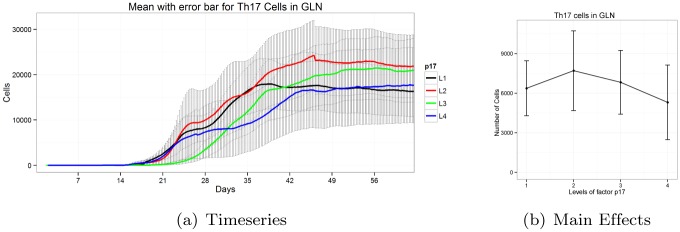
Timeseries and main effects of *Th*17 cells in GLN for parameter *p*_17_ exhibits bell shaped pattern.

The effects of parameters can vary dramatically based on the parameter levels. Fig 5 shows the effect of parameter *p*_17_, which is the probability that a resting T cell differentiates to *Th*17 with the presence of inflammatory phenotypes. In the figure, on the boundary conditions (Level 1 and Level 4) the average population of *Th*17 is lower than the population simulations for Levels 2 and 3. This can be explained by the fact that, at *p*_17_ = 0 (Level 1), according to the model, resting T cells do not produce *Th*17, instead only inflammatory *Th*1 cells are produced. *Th*17 is only produced from the transition of *iTreg* to *Th*17. For this reason, we observe a lower number of *Th*17 cells for Level 1. On the other hand, at *p*_17_ = 1 (Level 4) all resting T cells produce only *Th*17 cells but not *Th*1. But, this differentiation requires inflammatory phenotypes such as *Th*1. Since no *Th*1 is directly produced from resting T cells by the model, there is a lack of inflammatory phenotypes to make the transition from resting T to *Th*17. For this reason, a lower number of *Th*17 cells is observed. For the intermediate levels (Level 2 & 3) a higher number of *Th*17 cells is observed as there is a balance between the rate of resting T cells to *Th*17 cell transition and an adequate number of *Th*1 cells as activators. This depicts one of the complex behaviors of parameter levels, which are typically bell shaped or inverted bell shaped in the model output.

#### Sigmoid main effect patterns exhibit switching behavior

**Fig 6:**
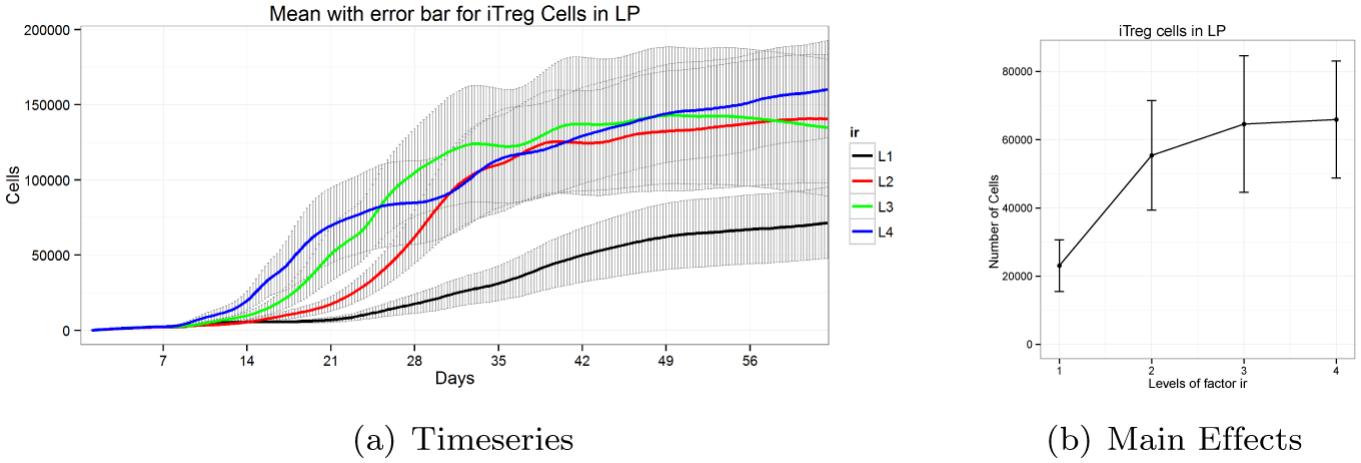
Timeseries and main effects of *iTreg* cells in LP for parameter *i*_*r*_ exhibits sigmoid pattern.

Minor differences in parameter levels play crucial roles in delineation of model output. For instance, in Fig 6, when the parameter *i*_*r*_’s value is changed from Level 1 to Level 2 we observe a dramatic change. However, we do not observe changes when Levels 3 or 4 are incremented. It appears that the effect of parameter value of *i*_*r*_ is saturated after Level 2. Hence the significant value of *i*_*r*_ must be within Levels 1 and 2. Parameter *i*_*r*_ is the inhibitory factor of *iTreg* to *Th*17 transition. The Treg population is predominant over the *Th*17 cells during *H. pylori* infection. Hence, for lower values, a slight change of *i*_*r*_ parameter has a significant effect on the system. Once the regulatory T cell population is significantly increased, the difference observed in these values is lower for values higher than 1, as the probability of transition from *iTreg* to *Th*17 is already very low. This kind of behavior results in a sigmoid main effect plot. Thus, a dramatic transition between two parameter values are observed; however, other values beyond the two levels do not have significant effect on the system output.

#### The behaviors of complex patterns are not well understood

**Fig 7:**
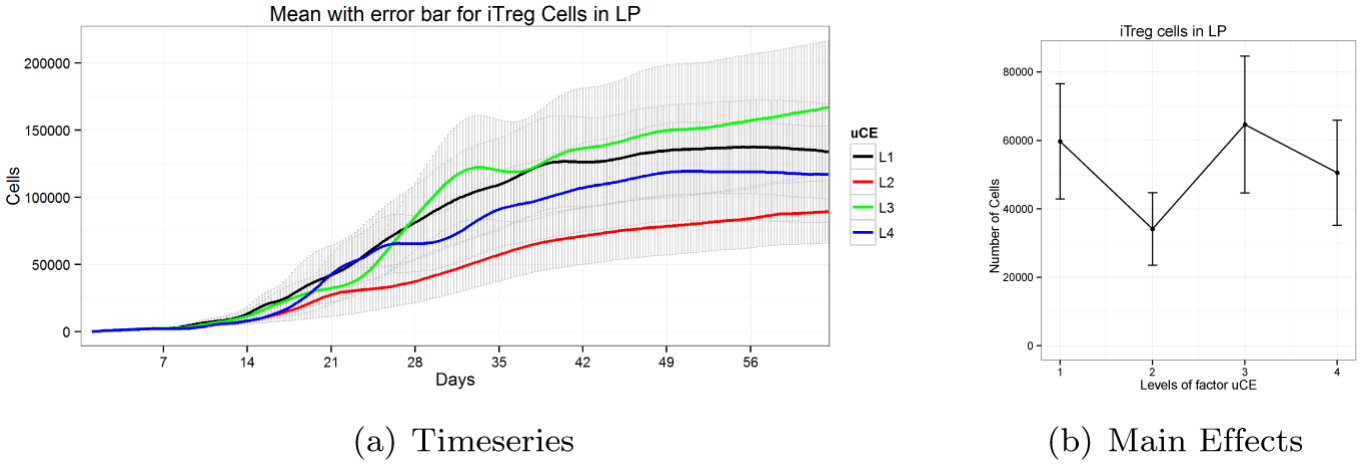
Timeseries and main effects of *iTreg* cells in the GLN for parameter *μ*_*CE*_ exhibits complex pattern.

A fourth pattern is also observed in this study. With the increase of parameter values from Level 1 to Level 4, the number of cells increases and decreases creating a zig-zag shaped pattern in the main effect plot (see Fig 7). For example, consider the effect of parameter *μ*_*CE*_ on *iTreg* cells in lamina propria. Parameter *μ*_*CE*_ is the probability that pro-inflammatory epithelial cells are damaged by the inflammatory factors. By moving from Level 1 to Level 2, it is observed that the population of *iTreg* cells falls drastically and rises again for Level 3. The cell population falls again for Level 4. This type of parameter behavior requires further investigation.

### ANOVA based analysis of global sensitivities

For the output immune responses and its 25 input parameters, it is important to investigate the underlying causes for how the parameters affect model outputs. To determine the impact of each parameter on the ENISI model, analysis of variance (ANOVA) was performed to find the most significant parameters. The key idea behind ANOVA use here is to link output and input through a statistical model with a normally distributed random error term. Based on the data, one can fit the model and quantify how the total variation of the output can be decomposed to variations contributed by estimated parameters, thus explaining the effects of sensitivity of input parameters.

Note that there are 128 different parameter setting for global sensitivity analysis, and each setting is replicated for 15 times in simulations. Therefore, there are 128 × 15 = 1920 data points for the ANOVA. For the output, the average count of each cell type over a time period (e.g., the entire time period or a time segmentation) as the response was used. In this study, by applying the ANOVA including the main effects, the observed variance in the output response is partitioned into components attributable to the 25 different parameters. Such an analysis provides a statistical quantification of whether or not each parameter plays a vital role to contribute the observed total variation of response output. Specifically, the significance of a parameter contributing to the total variation is examined by the statistical hypothesis testing using *F* statistic [76]. The *F* statistic is obtained by computing the ratio between the variation of output explained by the parameter and the total variation of response output. Thus, a large value of *F* statistic indicates a strong evidence of the significance of the parameter. The *p*-value based on the *F* test can be calculated accordingly [77], having small values with large *F* statistic. A statistically significant result with *p*-value lower than a user-defined threshold (i.e. significance level) justifies the rejection of the null hypothesis that the parameter is insignificant. Here the significant level is set to be 0.05. Recall that the design used for the experiment is an orthogonal array with 128 runs. Such a design makes each column of the design matrix mutually orthogonal to each other. As a result, the ANOVA based on such a design allows the estimated main effects to not correlate with each other. It provides an efficient technique to determine the sensitivity of each parameter with respect to the output of cell counts.

To ensure the validity of the ANOVA results, we also examine the model assumptions of the ANOVA approach. Note that the ANOVA method assumes that the independent and identically distributed error term exhibits normal distribution. For the global sensitivity analysis, we use the residual scatter plot to examine the independence of the data, and examine the normality assumption of the error terms through the histogram of residuals and the QQ-normal plot. The scatter plot, histogram, and QQ-normal plots of the residuals for the nine cells types as responses are shown in S1 Fig. As we can see from the figures, the independence and normality assumptions are generally valid when using the ANOVA for the global sensitivity analysis for our data. It is worth pointing out there are some potential outliers existing in the data, which could distort the model assumptions to some extent. Detailed investigation of those abnormal points with domain experts are often recommended before removing these points from further analysis.

#### Overall significance of modeling parameters

**Fig 8:**
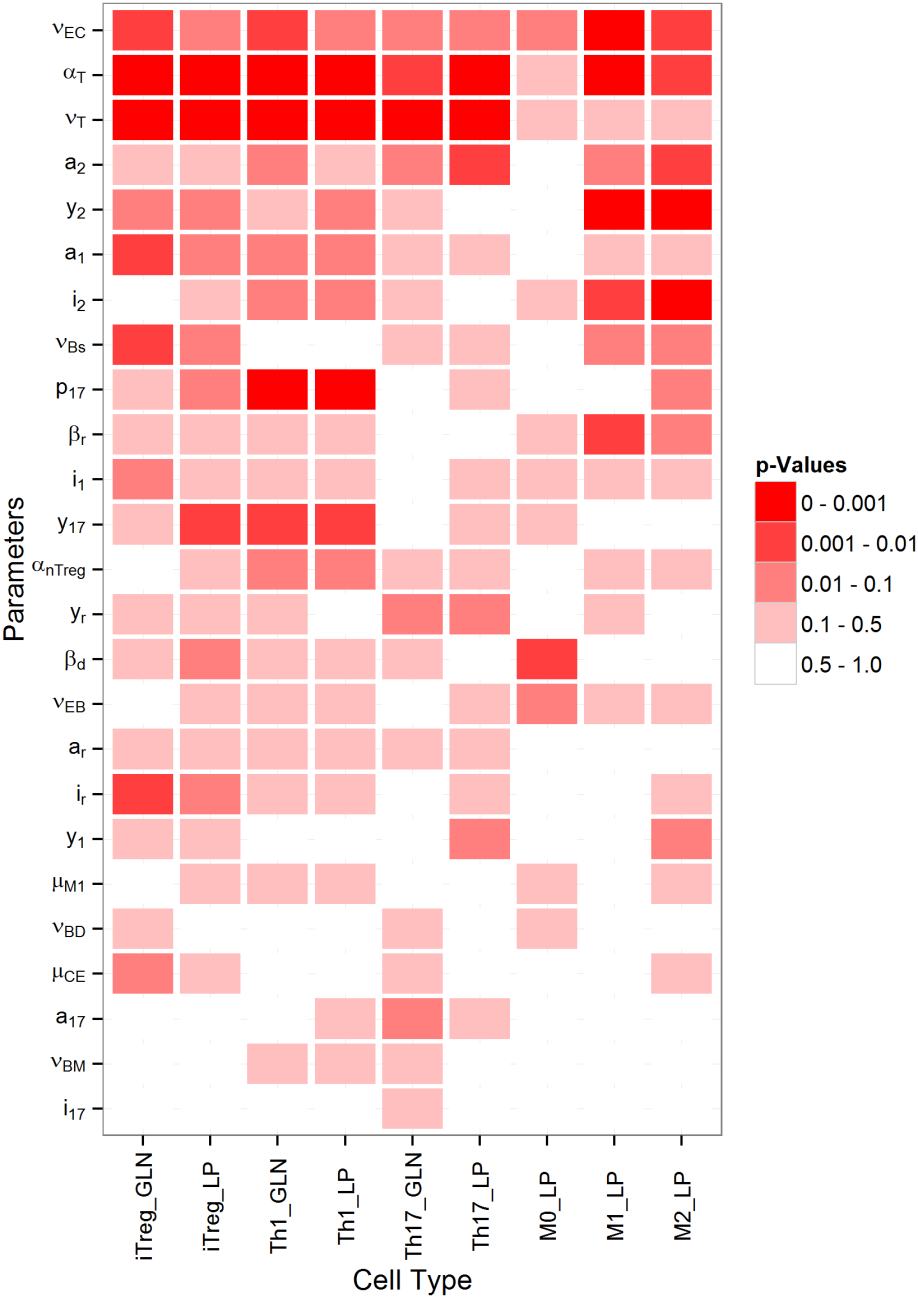
Overall *p*-values of modeling parameters on cell subsets in the synthetic gastric mucosa.

Using the technique described above, ANOVA analysis was performed to calculate the *p*-values of significance of a parameter for each of the nine output variables. This is shown in Fig 8. The x-axis represents the 25 parameters and the y-axis represents the nine output cell types.

From Fig 8 it is clear that some parameters, such as *a*_*T*_, *v*_*T*_, *v*_*EC*_, are significantly important for each cell type. There are certain parameters that are significant only for T cell populations (e.g. *p*_17_, *y*_17_) and some that are responsible for macrophages (e.g. *b*_*r*_).

The global sensitivity analysis shows which parameters are most important for model tuning. Hence, it is also helpful to identify the parameters that have the smallest effects on the model. For example it is clear that parameter *a*_17_ and *i*_17_ have little effect on the system.

#### Temporal significance of modeling parameters

In addition to measuring sensitivity analysis for a parameter over the entire time period, it is possible to analyze in which time period a parameter is most significant. This is particularly important because it allows investigation of the significance of a parameter over time.

**Fig 9:**
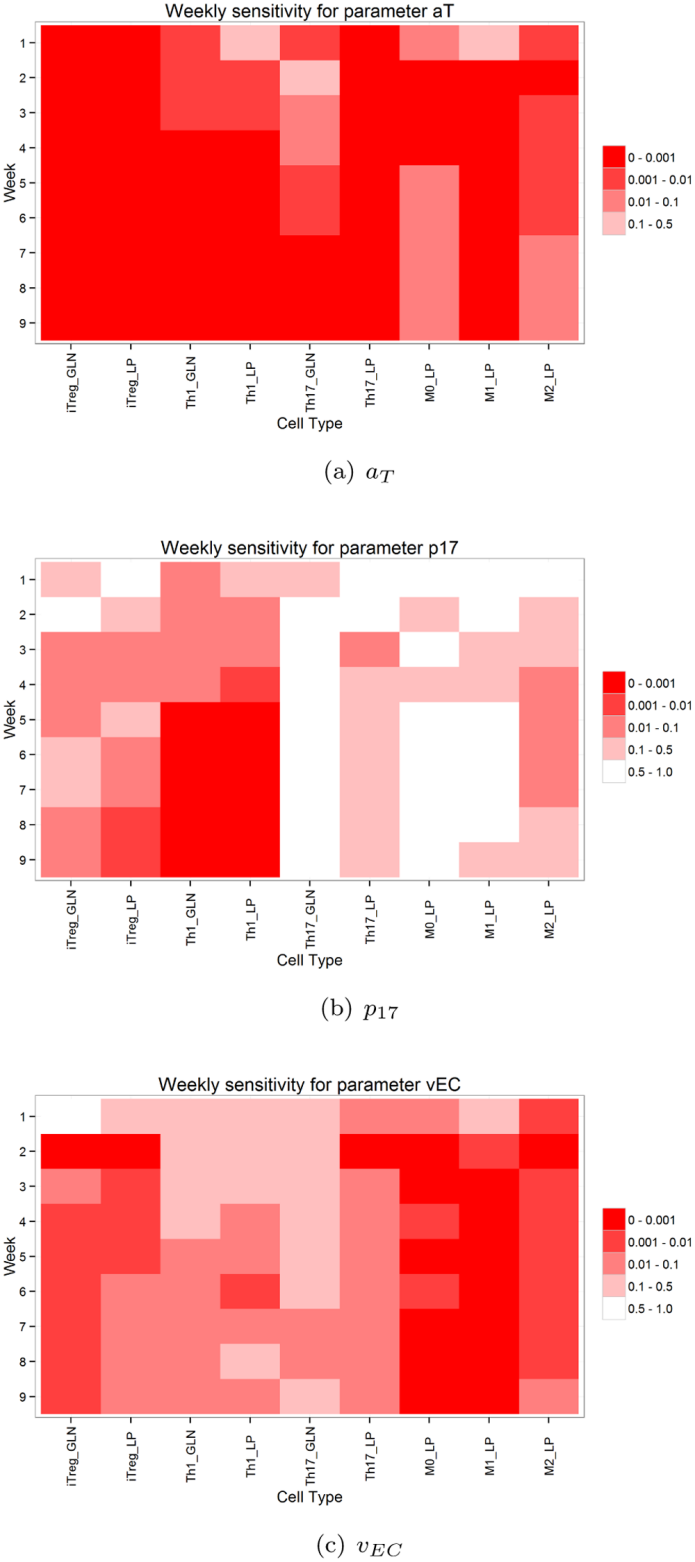
Temporal effects of parameters *a*_*T*_, *p*_17_ and *v*_*EC*_ on output cells type per week.

For this analysis we divided the entire time period into nine segments, each representing one experimental week. The one experimental week time interval is chosen from a biological point of view, as we are interested to see how the inflammation progresses per week. Also, in the *in vivo* experiments, biological data is collected weekly, which allows us to compare and validate simulated and biological results. Next, the significance of a parameter within the nine week period was plotted. The significance of a parameter is represented by the *p*-value for the changes of the parameter determined using ANOVA. The results for parameters *v*_*EC*_, *a*_*T*_ and *p*_17_ are given in Fig 9. Major changes in the dynamic significance of parameters were identified. In part the ANOVA findings are intuitive and serve as a measure to ensure that the model behaves in a biologically relevant manner. For instance, the probability of resting T cell stimulation, *a*_*T*_, has a high impact on all T cell subsets and activated macrophages with increasing significance over time. This is explained primarily by T cell population density during the course of infection. At the beginning of infection, *Th*1 and *Th*17 populations are low in contrast to when infection progresses, which is when these cell types become more critical for proliferation and differentiation of additional T cells in the gastric lamina propria (Fig 9).

One interesting finding is seen in the behavior of epithelial cells in response to inflammatory factors. Specifically, the probability that an epithelial cell transitions into a proinflammatory state as determined by parameter *v*_*EC*_ has a higher impact on overall immunity when compared to epithelial damage (*b*_*r*_). In fact, *v*_*EC*_ is the single most significant parameter in the model as determined by ANOVA. These results suggest that epithelial cells that come in contact with *H. pylori* significantly influence the overall immune response. Moreover, the temporal effects of *v*_*EC*_ increase as infection progresses and don’t approach the significant *p*-value bracket of 0.01–0.1 until at least 4 weeks post-infection, implying that this response wouldn’t be detected until late after infection (Fig 9c). Although out of the scope of this paper, a longer time course simulation should be performed to predict at which time frame this epithelial cell property is most crucial to host immunity to *H. pylori*.

#### Selection of most significant parameters

In order to refine parameter selection we need to determine the most significant parameters based on their impact on model dynamics. To rank the parameters we use two different systems using global sensitivity analysis. First we take average *p*-values for each of the output cell types and order them from smallest to largest. In this way, we find that the top ten significant parameters are: *ν*_*EC*_, *a*_*T*_, *ν*_*T*_, *a*_2_ *y*_2_, *a*_1_, *i*_2_, *ν*_*Bs*_, *p*_17_, and *b*_*r*_. We also ranked the parameters based on how many outputs are significant for each parameter. For this type of ranking we find that: *a*_*T*_, *ν*_*T*_, *ν*_*EC*_, *y*_2_, *p*_17_, *i*_2_, *y*_17_, *a*_2_, *a*_1_, and *ν*_*Bs*_ are the top ten significant parameters. Both lists are almost identical other than *b*_*r*_ and *y*_17_. Parameter *y*_17_ can be considered more important if *p*-values and the number of significant output parameters are compared.

### Local sensitivity analysis

Global sensitivity analysis measures the significance of each parameter. We can rank the most significant parameters based on *p*-values as described in the previous section. We choose the top ten significant parameters to analyze their local sensitivity behavior. The ten most significant parameters along with the corresponding test values are shown in Table 4.

**Table 4:** The list of parameter values used for mono-factorial sensitivity analysis.

Parameter	Value 1	Value 2	Value 3	Value 4
*a*_*T*_	0.5	0.7	0.8	1
*v*_*T*_	0.5	0.7	0.8	1
*v*_*EC*_	0.01	0.1	0.25	0.5
*y*_2_	0.1	1	4	10
*p*_17_	0.1	0.25	0.5	0.9
*i*_2_	0.1	1	4	10
*y*_17_	0.1	1	4	10
*a*_2_	0.1	11	10	5
*a*_1_	1	1	10	5
*v*_*Bs*_	0.05	0	1	0.5
*μ*_*M*1_	1	0.5	0.25	0.0

To study the effect of a single parameter over the population, we created a base set of parameter values called the base configuration from studies in literature and experimental calibration process. As the global sensitivity results show the region of interest, we selected three new different values from the region. Thus every parameter has four different values. Table 4 lists all the parameters and the tested values.

In the following sections, the local effects of the top two significant parameters *p*_17_ and *v*_*EC*_ along with a biologically interesting parameter *μ*_*M*1_ are described in detail.

#### Parameter *p*_17_ control the T cell dynamics significantly

**Fig 10:**
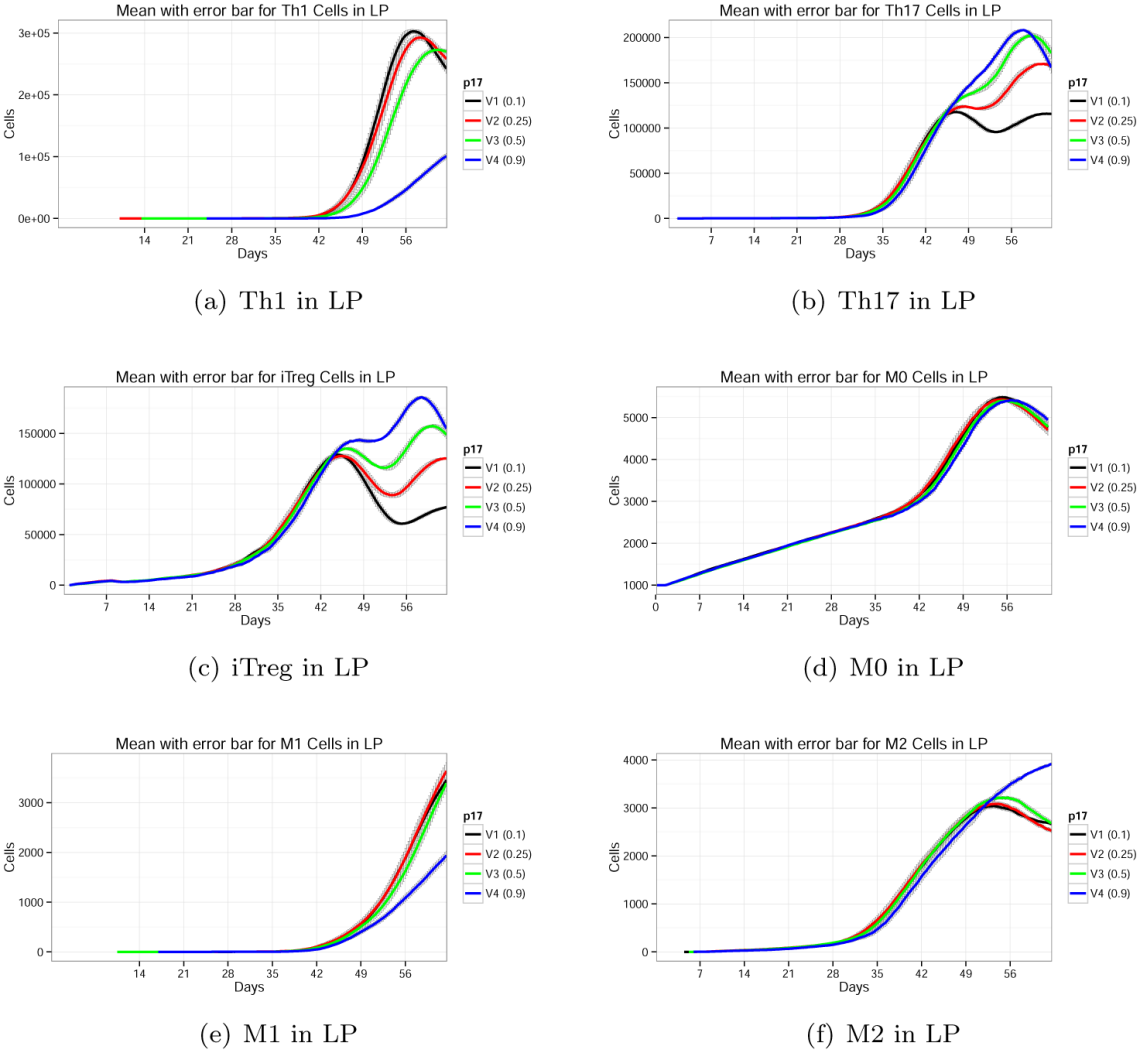
Effect of parameter *p*_17_ on *Th*1, *Th*17, *iTreg*, *M*0, *M*1, and *M*2 phenotypes.

Parameter *p*_17_ is the probability of resting T cell stimulation to *Th*17. The default value for the model is set at 0.1. We also observed the output for values of 0.25, 0.5, and 0.9. The experimental results are shown in Fig 10. When the value of *p*_17_ is increased, resting T cells are more likely to produce *Th*17*Prolif* than *Th*1*Prolif*. So, production of *Th*17 is increased. We can see that with increasing *p*_17_ we should create lower numbers of *Th*1 as compared to *Th*17. We also implemented transition of resting T to contact dependent transition. The transition depends on *eDC* and *M*1. As *p*_17_ does not directly affect *M*1 or *eDC*, we do not observe much change in the dynamics of *M*1 and *eDC* for *p*_17_. So, the effect of parameter *p*_17_ for the *Th*1, *Th*17 and *iTreg* dynamics are not very sensitive.

#### Parameter *v*_*EC*_ has very high sensitivity

**Fig 11:**
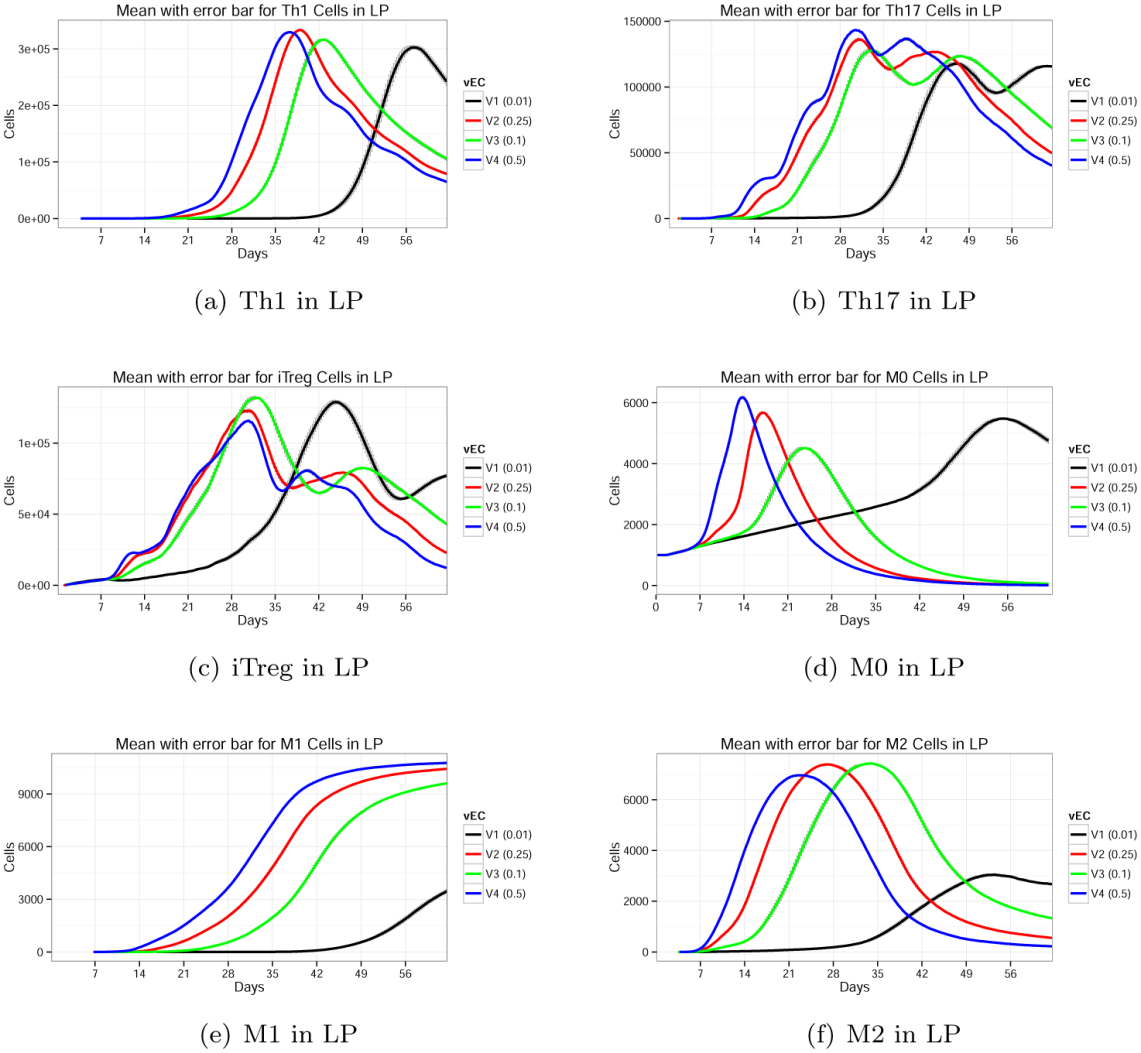
Effect of parameter *v*_*EC*_ on *Th*1, *Th*17, *iTreg*, *M*0, *M*1, and *M*2 phenotypes.

Parameter *v*_*EC*_ is the probability that EC cells transition into *pEcell*. The experimental results are shown in Fig 11. The default value of *v*_*EC*_ is 0.01. This is the lowest probability value of the parameter that was tested. When we increased the probability to a slightly higher value of 0.1, we observed significant change. Increasing *v*_*EC*_ from 0.01 to 0.1 increases *iTreg* cells at the GLN, and *M*0, *M*1, and *M*2 macrophages in the LP primarily, and exerts a lesser impact in *Th*1 and *Th*17 cell subsets. A recent paper implicates Sonic Hedgehog, a regulator of gastric epithelial cell differentiation and function, which is increased after *H. pylori* infection [78], as critical for macrophage recruitment to the stomach LP early after *H. pylori* infection [79].

#### Parameter *μ*_*M*1_ controls macrophage dynamics

**Fig 12:**
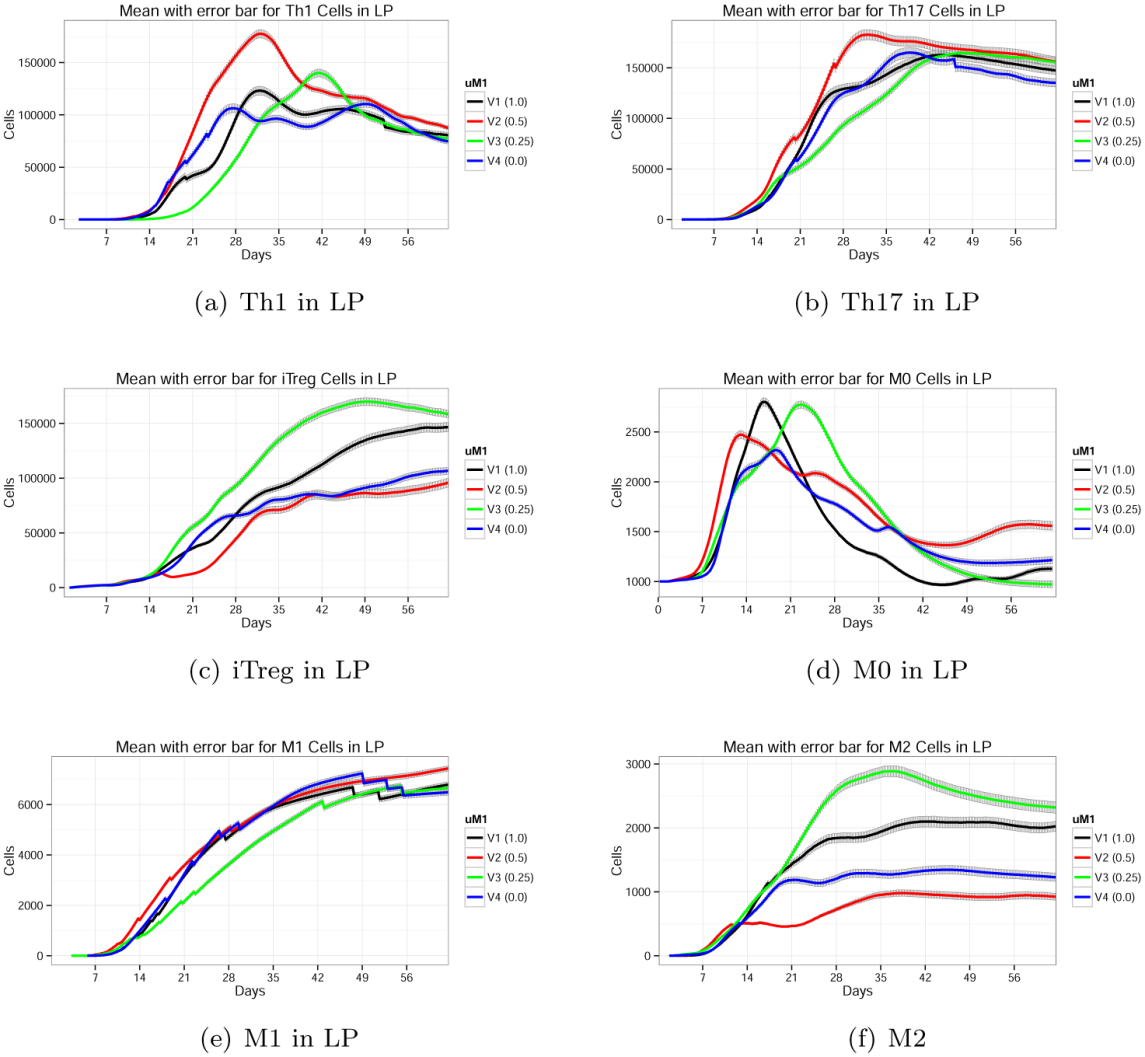
Effect of parameter *μ*_*M*1_ on *Th*1, *Th*17, *iTreg*, *M*0, *M*1, and *M*2 phenotypes.

Parameter *μ*_*M*1_ represents the ability for pro-inflammatory *M*1 cells to eliminate bacteria. The default value of *μ*_*M*1_ is 1. To observe the effect of *μ*_*M*1_ on the system, the value was changed to 0.5, 0.25 and 0.0 in decreasing order such that fewer bacteria are killed. The ability for macrophages to rid the system of *H. pylori* caused the most noticeable changes in a peak of lamina propria myeloid cells (*M*0) between days 10 and 22 (Fig 12). More than a 10-day delay occurs in the *M*0 population when *μ*_*M*1_ from is shifted from 0.25 to 0.5. Additionally, at the higher killing capacity (*μ*_*M*1_ = 0.5) bacteria is cleared more rapidly but causes more significant inflammation when compared to *μ*_*M*1_ = 0.25. When *μ*_*M*1_ is set to its default value, the myeloid population peaks between days 15 and 16 and prevents an overzealous cellular response.

### Interpretation of sensitivity analysis results as new hypothesis

Based on the sensitivity analysis, *ν*_*EC*_ is the most critical factor in the system’s dynamics. This finding indicates that perturbations induced by contact of *H. pylori* with epithelial cells are the major drivers of the events and reactions that characterize the immune response to the bacteria. The great majority of *H. pylori* in the gastric mucosa is found free-floating in the mucus layer and associated with the apical side of epithelial cells [80, 81]. Thus, the model recapitulates this principle and the sensitivity analysis indicates that the epithelium is the compartment which receives the major impact of the infection. An interesting and less intuitive finding of the model is the relationship between epithelium and macrophages. Based on analysis across *ν*_*EC*_ ranges, activation of epithelial cells by *H. pylori* leads to a sequential accumulation of macrophage precursors (*M*0) between days 0 and 14/21 post-infection. This first wave is followed by a subset of *M*2 with regulatory/suppressor role, which peaks between days 21 and 35 post-infection. Starting at day 45 there is a predominance of *M*1 pro-inflammatory macrophages. This sequence of changes is caused by increased probability of epithelial cells transitioning to a proinflammatory state, which should directly induce a proinflammatory macrophage. It will be interesting to screen for the presence of regulatory macrophages in the gastric mucosa following *H. pylori* infection and evaluate their role in the outcome of the infection.

## Discussion

Sensitivity analysis of a large scale agent-based model is challenging due to complex model structure and expensive computation needed to run a full factorial experiment *in silico*. The running time of the models prohibits the consideration of a full factorial combination of parameters for global sensitivity analysis. In this work, we have developed a novel and comprehensive sensitivity analysis by using an effective experimental design strategy. In spite of a large number of parameters and a continuous space of parameter values, the proposed method can capture the global nature of the system and gain proper knowledge of the significance of parameters on the system.

The proposed experimental methods tackle the challenges faced in obtaining meaningful results and proper insight into the model. The design of experiments only needs an economic run size in order to obtain the proper sensitivity analysis on a global scale. The results also showed that the design, with four levels for each parameter, captures some interesting main-effect shapes. We observed the various shapes of main-effect plots and classified them into four general categories. Meaningful explanations are provided to illustrate the aptness of these shapes in the ENISI model with validation from biological reality.

The global sensitivity analysis guided us as to the range of parameter values. The model was then calibrated to a base parameter setting using biological literature and findings from *in vivo* experiments [64]. To further fine tune the model, we performed local sensitivity analysis of the ten most important parameters. We have varied the values of the parameters around the base value to observe changes in output. Whenever the outputs were deemed closer to experimental results, the parameter value was updated. With a few iterations, we achieved a calibrated model of the mucosal immune responses to *H. pylori* infection.

In this study, the global sensitivity analysis of ENISI revealed four distinct patterns for the main effects of the input parameters. For the monotonic pattern for a parameter, its contribution to the output is easy to understand and can be controlled by simply increasing or decreasing the parameter value. Bell shaped patterns reveal complex inter-dependency among more than one parameter. Also the complex zig-zag shaped patterns for a parameter are not yet well understood and require further investigation. Future work can investigate more on inter-dependencies and relationships among the parameters. Orthogonal arrays provided a natural way to capture pairwise interactions. The interaction effect plots can show the interaction of parameters with the system and their interdependencies. If the focus of the study is on three-way or higher order interactions, one can consider orthogonal arrays with a strength larger than two or some continuous-based design such as Latin hypercube [82, 83].

In this work, the experiment and analysis are performed on the *H. pylori* model implemented using the ENISI platform, an agent-based model. However, the proposed methodology is general in nature, and can work for other complex simulation systems with a large number modeling parameters, such as models in computational epidemics, social network analysis, etc. The proposed method can also be a useful tool for validation and parameter estimation with a limited budget in terms of experiment time and effort. The temporal sensitivity analysis also reveals the time periods when the system is sensitive to other parameters. To calibrate the computational model with some existing systems, it can be useful to collect experimental data in the specific time period of interest.

## Supporting Information

S1 Text Formalization of ENISI MSM. (PDF)

S1 Fig Residual scatter, histogram, and QQ-normal plots for nine output parameters. (TIF)

S1 File Values of parameters for each of the four levels. (XLSX)

S2 File Orthogonal array of 128 experiment runs on 25 factors. (XLSX)
