# Supplementary material for: Sensitivity Analysis of an ENteric Immunity SImulator (ENISI)-Based Model of Immune Responses to Helicobacter pylori Infection
Source: PLoS One. 2015 Sep 1;10(9):e0136139. doi: 10.1371/journal.pone.0136139 (PMC4556515; doi:10.1371/journal.pone.0136139)
Supplement: S1 Text — (PDF) [file pone.0136139.s001.pdf]

Formalization of ENISI MSM

Formalization of ENISI MSM

The underlying formal mathematical model for ENISI is a Co-evolving Graphical Discrete Dynamical System (CGDDS) framework [2, 1]. CGDDS is a time varying network model which captures the co-evolution of regulatory and effector mucosal immune responses, the inflammation/regulation dynamics, and individual cellular behavior. CGDDS is described in more detail in [1]. This mathematical model is extended in ENISI to capture the cellular interactions that occur with the movement of immune cells and bacteria. Here we provide an informal description of the mathematical model. For a comprehensive description please refer to [3].

An extended **Co-evolving Graphical Discrete Dynamical System** $CGDDS$ over a given domain D of state values is a triple $(G_t, \mathcal{F}, W)$ where G_t is a time varying graph, $\mathcal{F}$ is a set of state transition functions and W is an update scheme [4, 3]. In the rest of this paper, we will assume that nodes are updated synchronously. The components of CGDDS can be described as follows.

The dynamic network $G_t(V_t, E_t)$ is a graph where vertices and edges can change over time. For the sake of simplicity, we will ignore time index. The set of vertices $V = \{v_1, v_2, \ldots v_n\}$ represents the set of immune cells or bacteria. Also let $S = \{S_1, S_2, \ldots S_m\} \in D$ be a set of all possible states (phenotypes) that an individual cell can take. We also define $s_t(v) \in S$ as the state of any vertex $v \in V$ at time t.

The interactions between the vertices of the graph are based on spatial proximity. As cells move, their contacts are changed, which results in the addition or deletion of edges. These are the basis of changes within the contact network.

For each vertex (cell) v, g denotes the edge modification function that takes as input the state of the vertex $s_t(v)$ at time t and returns the set of

edges that v will be adjacent to in a given time period. In other words, g captures the time varying edges at $t+1$ in the graph.

Given this defined formalism, the cell state transitions occur as described in the following section. For each vertex $v \in V$ let f be the function representing state transition. The function f takes as input the state of the vertex at time t, the states of other vertices of the edges incident on v and provides the state of v at time $t+1$ as output. If vertices $v_1, v_2, \ldots, v_k$ are adjacent to vertex v we can define the state transition function for v as:

$$s_{t+1}(v) = f(s_t(v), s_t(v_1), s_t(v_2), \ldots, s_t(v_k))$$

This function may also be stochastic. At any time t, the configuration $\xi(t)$ of a CGDDS is defined as a vector $(s_t(v_1), s_t(v_2), \ldots, s_t(v_n))$, the states of all vertices. The time evolution of a CGDDS is represented by the sequence of successive configuration changes of the dynamic network.

References

[1] C. L. Barrett, K. Bisset, J. Chen, S. Eubank, B. Lewis, V. S. A. Kumar, M. V. Marathe, and H. S. Mortveit. Interactions among human behavior, social networks, and societal infrastructures: A case study in computational epidemiology. In S. S. Ravi and S. K. Shukla, editors, *Fundamental Problems in Computing*, pages 477–507. Springer Netherlands, 2009.

[2] C. L. Barrett, K. R. Bisset, S. G. Eubank, X. Feng, and M. V. Marathe. Episimdemics: an efficient algorithm for simulating the spread of infectious disease over large realistic social networks. In *Proceedings of the 2008 ACM/IEEE conference on Supercomputing*, SC '08, pages 37:1–37:12, Piscataway, NJ, USA, 2008. IEEE Press.

[3] K. Wendelsdorf, J. Bassaganya-Riera, K. Bisset, S. Eubank, R. Hontecillas, and M. Marathe. Enteric immunity simulator: A tool for in silico study of gut immunopathologies. *Proceedings of the IEEE International Conference Bioinformatics and Biomedicine*, 2011.

[4] K. Wendelsdorf, J. Bassaganya-Riera, R. Hontecillas, and S. Eubank. Model of colonic inflammation: Immune modulatory mechanisms in inflammatory bowel disease. *Journal of Theoretical Biology*, 2010.
