# Supplementary figures and images for: Sensitivity Analysis of an ENteric Immunity SImulator (ENISI)-Based Model of Immune Responses to Helicobacter pylori Infection
Source: PLoS One. 2015 Sep 1;10(9):e0136139. doi: 10.1371/journal.pone.0136139 (PMC4556515; doi:10.1371/journal.pone.0136139)

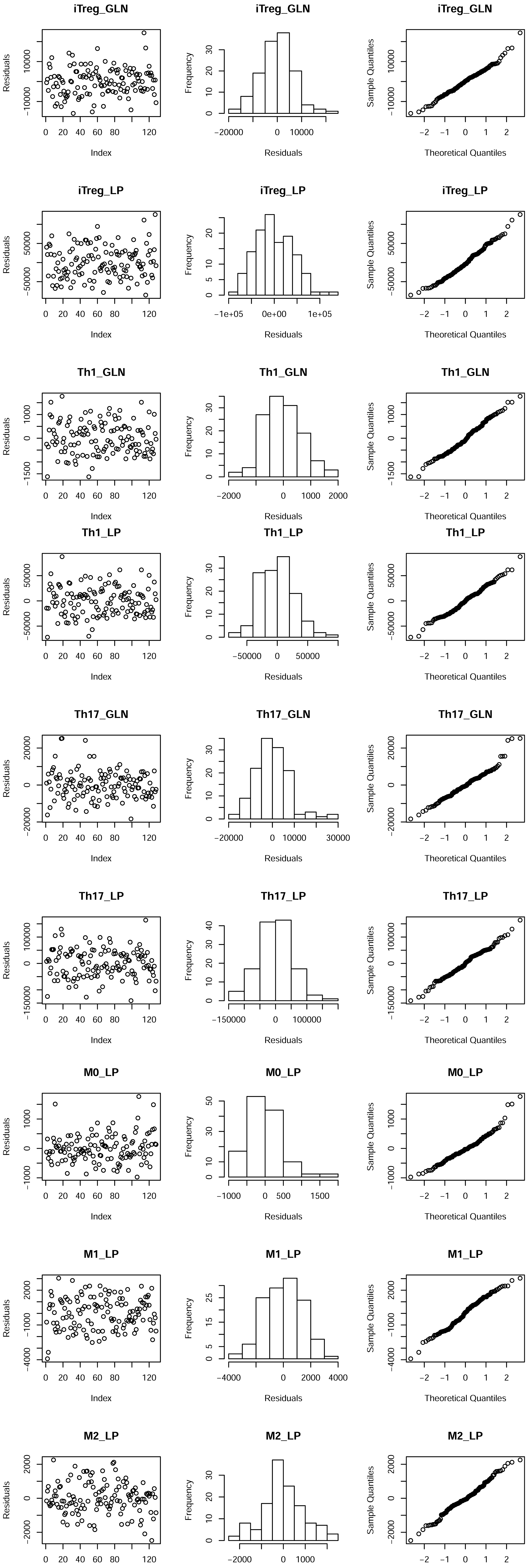

Supplement: S1 Fig — (TIF) [file pone.0136139.s002.tif]
